# Intake of herring oil, but not of anchovy oil, resulted in a lower serum cholesterol concentration in male Zucker Diabetic Sprague Dawley rats

**DOI:** 10.1017/S0007114524001454

**Published:** 2024-09-14

**Authors:** Eira V. Rimmen, Svein Are Mjøs, Eirik Søfteland, Oddrun A. Gudbrandsen

**Affiliations:** 1 Dietary Protein Research Group, Centre for Nutrition, Department of Clinical Medicine, University of Bergen, Bergen 5021, Norway; 2 Department of Chemistry, University of Bergen, 5020 Bergen, Norway; 3 Department of Medicine, Haukeland University Hospital, Bergen, Norway

**Keywords:** Cetoleic acid, Cholesterol, Lipoproteins, Diabetes

## Abstract

Patients with type 2 diabetes have increased risks for dyslipidaemia and subsequently for developing vascular complications. A recent meta-analysis found that cetoleic acid (C22:1n-11) rich fish oils resulted in lower cholesterol concentration in rodents. The aim was to investigate the effect of consuming fish oils with or without cetoleic acid on serum cholesterol concentration in diabetic rats and to elucidate any effects on cholesterol metabolism. Eighteen male Zucker Diabetic Sprague Dawley rats were fed diets containing herring oil (HERO) or anchovy oil (ANCO) or a control diet with soyabean oil for 5 weeks. The HERO diet contained 0·70 % cetoleic acid, with no cetoleic acid in the ANCO diet. The HERO and ANCO diets contained 0·35 and 0·37 wt% EPA + DHA, respectively. Data were analysed using one-way ANOVA. The serum total cholesterol concentration was 14 % lower in the HERO group compared with ANCO and Control groups (*P* = 0·023). The HERO group had a higher faecal excretion of bile acids (*P* = 0·0036), but the cholesterol production in the liver, the hepatic secretion of VLDL and the liver’s capacity to take up cholesterol were similar to controls. The ANCO diet did not affect the serum cholesterol concentration, but the hepatic cholesterol biosynthesis, the clearance of lipoprotein cholesterol and the excretion of bile acids in faeces were higher than in the Control group. To conclude, consumption of herring oil, but not of anchovy oil, led to a lower cholesterol concentration in a type 2 diabetes rat model.

High serum/plasma cholesterol concentration is a major risk factor for CVD^(1,2)^, which is the leading cause of disease burden globally affecting more than half a billion people annually^(3)^. Patients with type 2 diabetes (T2D) have an elevated risk for developing metabolic disturbances leading to dyslipidaemia and thus have an increased probability for CVD^(4–6)^. Treatment strategies for patients with T2D include lifestyle modifications comprising changes in dietary habits in order to reduce adiposity in patients with obesity and management of hyperglycaemia^(7)^. Strategies for reducing CVD risk include lowering of cholesterol concentration through lifestyle modifications and use of lipid-lowering drugs^(8)^.

Consumption of fish has been associated with a lower CVD risk in several studies^(9–11)^, and epidemiological studies suggest that intake of fish may protect against T2D^(12–14)^. The beneficial health effects of consuming fish have traditionally been ascribed to the long-chain PUFA EPA (C20:5n-3) and DHA (C22:6n-3)^(15)^, although consumption of fish oils or concentrates with high EPA and DHA contents does not affect the cholesterol concentration in humans^(16–19)^ and lowers the cholesterol concentration in rats and mice^(20)^ only when given in very high doses. Fish oils contain a plethora of fatty acids besides EPA and DHA, and in recent years, increased focus has been on the long-chain MUFA cetoleic acid (C22:1n-11). Cetoleic acid is found in high amounts in oils from certain fish species such as herring, which has relatively low contents of both EPA and DHA.

The Zucker Diabetic Sprague Dawley (ZDSD) rat model was generated by crossing the Zucker Diabetic Fatty (Lean +/+) with Sprague Dawley Crl:CD rats^(21)^. The ZDSD rat has an intact leptin signalling pathway and develops polygenetic metabolic disturbances with insulin resistance resulting in T2D, which progresses similarly to the disease in humans including the destruction of pancreatic *β*-islet cells^(21,22)^. The overt diabetes in these rats leads to dyslipidaemia and nephropathy^(22)^.

We have recently summarised and meta-analysed the available literature that investigates the effects of diets containing fish oils or fish oil concentrates that have a high content of cetoleic acid but low or no content of EPA and DHA on cholesterol concentration in rodents, showing that cetoleic acid-rich fish oils and concentrates prevent high cholesterol concentration^(23)^. However, none of the articles included in the systematic review provided any details on the mechanism(s) behind the lower cholesterol concentration after cetoleic acid intake^(23)^. Therefore, the main aim of the present study was to investigate the effect of consuming diets containing fish oils with or without cetoleic acid on the total circulating cholesterol concentration in ZDSD rats. The secondary aim was to investigate any effects on circulating markers of cholesterol metabolism as well as on hepatic enzymes and receptors central for cholesterol metabolism in these rats. Our hypothesis was that a diet containing herring oil (HERO) would result in a lower serum cholesterol concentration in ZDSD rats through changes in the hepatic pathways involved in cholesterol metabolism.

## Methods

### Animals and diets

Twenty-four male ZDSD/PcoCrl rats were obtained from Charles River. The rats were 81–95 d old at arrival (the exact date of birth was not provided by the breeder) and were housed in pairs in 1500U Eurostandard Type IV S cages (IVC Blue Line) with temperatures 22–23°C, in a room with controlled light/dark cycle (dark 20.00–06.00). The rats were fed standard chow as used in our animal facility (V1536, containing 19·1 % protein, 3·6 % fat, 4·8 % sugar, from ssniff Spezialdiäten GmbH) until they were approximately 16 weeks of age.

It is recommended by Charles River to feed the rats Purina LabDiet 5008 (23·6 wt% protein, 7·9 wt% fat, 2·7 wt% sucrose) until they are 16 weeks old and then switch to a higher fat diet to synchronise the onset of T2D in male ZDSD rats^(24)^. Charles River suggests using either Purina 5SCA/TestDiet5SCA (10·5 wt% protein, 25·6 wt% fat, 50·6 wt% carbohydrates) or Research Diets 12 468 (12·0 wt% protein, 25·5 wt% fat, 51·0 wt% carbohydrates) when rats are 16–19 weeks old and then switch back to the Purina LabDiet 5008. These higher fat diets have low protein contents, and the latter diet contains little essential fatty acids; therefore, an in-house diet was designed for the present study, containing 20 wt% protein (casein), 10 wt% sucrose, 16·2 wt% maltose dextrin, 32 wt% lard and 7 wt% soyabean oil. The detailed composition of this high-fat diet is presented in Table 1. The rats were fed this semi-purified high-fat diet until T2D had developed (blood glucose > 13·9 mmol/l).

**Table 1.** Compositions of the experimental diets and energy contents

Contents	High-fat diet (run-in diet)	Control diet	HERO diet	ANCO diet
Soyabean oil (g/100 g diet)	7·00	10·00	6·30	8·40
Lard (g/100 g diet)	32·00	–	–	–
Herring oil (g/100 g diet)	–	–	3·70	–
Anchovy oil (g/100 g diet)	–	–	–	1·60
Casein* (g/100 g diet)	21·76	21·56	21·56	21·56
Sucrose (g/100 g diet)	7·50	9·00	9·00	9·00
Maltose dextrin (g/100 g diet)	16·20	–	–	–
Maize starch (g/100 g diet)	4·33	48·23	48·23	48·23
Cellulose (g/100 g diet)	5·00	5·00	5·00	5·00
t-Butylhydroquinone (g/100 g diet)	0·0014	0·0014	0·0014	0·0014
Mineral mix (AIN-93-MX)† (g/100 g diet)	3·50	3·50	3·50	3·50
Vitamin mix (AIN-93-VX)‡ (g/100 g diet)	1·00	1·00	1·00	1·00
Growth and maintenance supplement§ (g/100 g diet)	1·00	1·00	1·00	1·00
l-Methionine (g/100 g diet)	0·16	0·16	0·16	0·16
l-Cystine (g/100 g diet)	0·30	0·30	0·30	0·30
Choline bitartrate|| (g/100 g diet)	0·25	0·25	0·25	0·25
Energy (kJ/g diet)	25·4	19·0	19·1	19·0

HERO, herring oil; ANCO, anchovy oil.

* Contains 91·9 % crude protein (high-fat diet) or 92·78 % crude protein (other diets).

† Contains sucrose (221 g/kg).

‡ Contains sucrose (967 g/kg).

§ Contains vitamin B_12_ (40 mg/kg) and vitamin K_1_ (25 mg/kg) mixed with sucrose (995 g/kg) and dextrose (5 g/kg).

|| Contains 41 % choline.

When diabetes was established, the rats were randomly assigned to receive one of the three experimental diets by drawing paper lots from a jar, each group consisting of six rats. The rats were given random numbers that could not be linked to the experimental group. The rat cages were randomly placed in the rack. The experimental semi-purified diets were modified versions of the American Institute of Nutrition’s recommendation for growing laboratory rodents (AIN-93G)^(25)^ with the addition of 1·6 g methionine/kg diet as recommended by Reeves^(26)^ and differed only in their lipid sources (Table 1). All diets contained 20 wt% protein (casein), 10 wt% fat and 11·8 wt% sucrose and had comparable energy content. We chose to use the AIN-93G diet instead of the AIN-93M diet for maintenance containing 15 wt% protein since the ZDSD rats will develop insulin resistance. Insulin resistance leads to increased muscle protein breakdown in both rodents and humans^(27,28)^. The two intervention diets contained either refined oil prepared from herring (*Clupea harengus*) residuals (the HERO diet) or refined oil prepared from whole anchovies (Engraulidae) (the ANCO diet), designed with a comparable content of EPA + DHA but with different cetoleic acid content in the diets. All diets contained adequate amounts of essential fatty acids according to Reeves *et al.* ^(25)^. The control diet contained soyabean oil as the only lipid source. All ingredients were purchased from Dyets Inc. except casein, which was purchased from Sigma-Aldrich, HERO from Pelagia AS and anchovy oil from Epax Norway AS. The rats had *ad libitum* access to feed and water in their home cage. The diets were stored at –15°C, and daily portions were thawed in the morning.

### Design

All rats were weighed three times per week. The water intake was recorded daily for all cages to detect any abrupt increase in drinking, as this would indicate the development of diabetes and/or failing kidney function. Non-fasting glucose concentration was measured in the morning (before 08.00) of the first and the last day of high-fat feeding, using blood from the dorsal tail vein and a blood glucose measuring device (Contour next; Bayer Consumer Care AG). After 30–31 d, the rats were fasted for 6 h for measurement of glucose in the blood from the dorsal tail vein using Bayer Contour’ next blood glucose measuring device. After 34–35 d of feeding the experimental powder diets, the rats were housed individually in IVC-4 for 24 h for collection of faeces and measurement of feed and water intake, without fasting in advance, in cages equipped with one Fat Rat Hut and softwood bedding, with ad libitum access to powder feed in a ceramic bowl and a water bottle. Faeces were carefully collected and frozen at –80°C until analysis. At the end of the experimental period, that is, after 36–37 d with powder feed, the feed was withdrawn at 06.30, and rats were placed individually in metabolic cages (Ancare Corp.) for 30–40 min for collection of ≥ 1 ml urine. Rats were fasted for a total of 6 h, with free access to drinking water, and were euthanised while anaesthetised with isoflurane (Isoba vet, Intervet, Schering-Plough Animal Health) mixed with oxygen. The body length was measured with a ruler, while the rats were anaesthetised. Blood was drawn from the heart and collected in BD Vacutainer SST II Advance gel tubes (Becton, Dickinson and Company) for isolation of serum, and serum was frozen at –80°C. The liver and the epididymal white adipose tissues from both sides were carefully dissected out and weighed, and frozen at –80°C. Kidneys and the heart was dissected out and frozen at –80°C.

The personnel handling the rats and conducting the analyses were blinded to the rats’ group allocation. The rats were handled and euthanised in random order.

### Environmental enrichments

The individually ventilated cages (IVCs) were equipped with one gnawing block (Aspen brick, size 100 mm × 20 mm × 20 mm, TAPVEI® Harjumaa, Estonia OÜ), three paper sachets containing softwood bedding for nesting material (2HK Nestpak, Datesand Ltd) and one red polycarbonate hut (Fat Rat Hut, size 150 mm × 165 mm × 85 mm, Datesand). The rats showed signs of behavioural changes during the second week of housing in IVC, and it was therefore decided to try periodic stays in a large ‘playcage’ with the purpose to increase their well-being and to provide cognitive as well as physical stimulation. This has been described in detail elsewhere^(29)^.

### Analyses of diets

Lipids in the diets were extracted as described by Bligh and Dyer^(30)^. Fatty acids in the lipid extracts were quantified by GC after lipid extraction and methylation, as described previously^(31)^. Cholesterol in the lipid extracts was measured after evaporation to dryness using nitrogen and re-dissolving in isopropanol before quantification on the Cobas c111 system (Roche Diagnostics GmbH) using the CHOL2 (Cholesterol Gen.2) kit from Roche Diagnostics. The dietary caloric content was measured by Nofima BioLab by a bomb calorimeter method in accordance with ISO9831:1998^(32)^.

### Analyses in serum

Serum total cholesterol, LDL-cholesterol, HDL-cholesterol and TAG were quantified on the Cobas c111 system (Roche Diagnostics) using the CHOL2 (Cholesterol Gen.2), LDLC3 (LDL-Cholesterol Gen.3), HDLC4 (HDL-Cholesterol Gen. 4) and TRIGL (Triglycerides) kits from Roche Diagnostics. Bile acids were quantified by using the Total Bile Acid Assay Kit (Diazyme Laboratories, Inc.) on the Cobas c111 system. Free cholesterol concentration was measured using the Cholesterol/Cholesteryl Ester Assay Kit (ab65359, from Abcam) read at 570 nm on a SpectraMax Plus384 Microplate Reader (Molecular Devices). The cholesteryl ester concentration was calculated as the difference between total and free cholesterol. All samples for each of the analytes were analysed on the same day (and on the same 96-well plate for free cholesterol quantification), and the CV were below 6 % for all colorimetric assays.

Apolipoprotein B48 was measured using the MBS753664 Rat Apolipoprotein B48 ELISA Kit (MyBioSource Inc.) and apolipoprotein B100 was measured using the MBS723231 Rat Apolipoprotein B100 ELISA Kit (MyBioSource). Lecithin-cholesterol acyltransferase (LCAT) was measured using the LS-F34827 Rat LCAT ELISA Kit (LifeSpan BioSciences, Inc.). Plates were read at 450 nm on a SpectraMax Plus384 Microplate Reader. All samples were analysed simultaneously in the same plate from each of the ELISA assays, with CV < 5 %.

### Analyses in urine

Glucose and carbamide were measured in urine on the Cobas c111 system (Roche Diagnostics) using the GLUC2 (Glucose HK) and UREAL (Urea/BUN) kits from Roche Diagnostics. All samples for each of the analytes were analysed on the same day, with CV < 3 %. The 24 h excretions of glucose and carbamide in urine were estimated based on the assumption that the volume of urine produced was similar to the water intake during 24 h.

### Lipids in the liver, kidney and heart

Lipids were extracted from the liver, kidney and heart using a mixture of methanol and chloroform, as described by Bligh and Dyer^(30)^. The lipid extracts were evaporated to dryness under nitrogen and re-dissolved in isopropanol before quantification of total cholesterol and TAG on the Cobas c111 system using the CHOL2 (Cholesterol Gen.2) and TRIGL (Triglycerides) kits from Roche Diagnostics.

### Protein analyses in the liver

Liver samples were homogenised in PBS, and liver protein was quantified with the Bradford dye-binding method^(33)^ using a protein assay dye reagent (Bio-Rad Laboratories) with bovine serum albumin (Bio-Rad Protein Assay Standard II, Bio-Rad Laboratories) as the standard. 3-Hydroxy-3-methylglutaryl CoA (HMG-CoA) reductase was measured using the rat HMG-CoA reductase/HMGCR ELISA Kit (Sandwich ELISA), cat no. LS-F15758 (LifeSpan BioSciences, Inc.). LDL receptor was measured using the Rat LDLR/LDL Receptor ELISA Kit (Sandwich ELISA) cat no. LS-F11934 (LifeSpan BioSciences, Inc.). Cholesterol 7 *α*-hydroxylase (CYP7A1) was measured using the Rat CYP7A1 ELISA Kit (Sandwich ELISA), cat no. LS-F9946 (LifeSpan BioSciences, Inc.). Scavenger receptor class B, member 1 (SCARB-1) was measured using the Rat SCARB-1/SR-BI ELISA Kit (Sandwich ELISA), cat no. LS-F23258 (LifeSpan BioSciences, Inc.). Proprotein convertase subtilisin/kexin type 9 (PCSK9) was measured using the Rat PCSK9 ELISA Kit (Sandwich ELISA), cat no. LS-F9168 (LifeSpan BioSciences, Inc.). Acetyl-CoA carboxylase (ACC) was measured using the Rat ACC ELISA Kit (Sandwich ELISA), cat no. LS-F35376 (LifeSpan BioSciences, Inc.). Diacylglycerol O-acyltransferase 2 was measured using the Rat DGAT2 ELISA Kit, cat no. EKX-MN0QK8 (Nordic Biosite). Microsomal TAG transfer protein (MTTP) was measured using the Rat MTTP/MTP ELISA Kit (Sandwich ELISA), cat no. LS-F25381 (LifeSpan BioSciences, Inc.). Solute carrier family 2 (facilitated GLUT), member 2 (SLC2A2) was measured using the Rat SLC2A2/GLUT2 ELISA Kit (Sandwich ELISA), cat no. LS-F25346 (LifeSpan BioSciences, Inc.). Carnitine palmitoyltransferase 1A (CPT-1A) was measured using the Rat CPT-1A ELISA Kit (Sandwich ELISA), cat no. LS-F33196 (LifeSpan BioSciences, Inc.). Plates were read at 450 nm on a SpectraMax Plus384 Microplate Reader. All samples were analysed simultaneously in the same plate for each of the ELISA assays, with CV < 5 %. Concentrations of HMG-CoA reductase, LDL receptor, CYP7A1, SCARB-1, PCSK9, ACC, DGAT2, MTTP, SLC2A2 and CPT-1A in the liver are presented relative to protein.

### Analyses in faeces

Lipids in faeces were extracted using a mixture of methanol and chloroform, as described by Bligh and Dyer^(30)^. The lipid extracts were evaporated to dryness under nitrogen and re-dissolved in isopropanol before quantification of total cholesterol on the Cobas c111 system using the CHOL2 (Cholesterol Gen.2) kit from Roche Diagnostics.

Faecal samples were freeze-dried, and total faecal bile acids were quantified using the method described by Suckling *et al.* ^(34)^ using Chromabond C18 ec (3 ml/200 mg, Macherey-Nagel) and the Total Bile Acid Assay Kit (Diazyme Laboratories, Inc.) on the Cobas c111 system.

### Outcome measurements

The primary outcome was to investigate the effect of consuming diets containing fish oils with or without cetoleic acid on the total circulating cholesterol concentration in ZDSD rats. The secondary aim was to investigate any effects on circulating markers of cholesterol metabolism as well as on hepatic enzymes and receptors central to cholesterol metabolism in these rats.

### Statistical analyses

This is the first study to investigate the effects of fish oil consumption in ZDSD rats; therefore, data on effect size were not available for sample size calculation or minimally detectable effect sizes. Based on studies conducted in rats and mice using cetoleic acid-rich fish oils with group sizes of six to twelve rodents/group^(23)^, we designed this study with eight rats per experimental group. We expected all rats to become diabetic when fed the high-fat diet; however, 25 % of the rats did not develop diabetes. Therefore, statistical analyses are conducted with *n* 6 rats in each experimental group.

Statistical analyses were conducted using SPSS Statistics version 28 (SPSS, Inc., IBM Company). All data were evaluated for normality using the Shapiro–Wilk test, revealing that most variables were normally distributed; therefore, one-way ANOVA was used to compare the experimental groups. Since this study is regarded as an exploratory study without the possibility of a proper calculation of the necessary sample size, when appropriate, the ANOVA analyses were followed by the Tukey HSD *post hoc* test as recommended by Lee *et al.* ^(35)^. The cutoff value for statistical significance was set at a probability of 0·05.

## Results

### Description of diets

The experimental powder diets had similar energy content of around 19 kJ/g diet and differed only in their lipid source (Table 1). The content of EPA + DHA was comparable in the HERO diet and the ANCO diet, whereas the long-chain MUFA cetoleic acid was found only in the HERO diet. EPA, DHA and cetoleic acid were not detected in the control diet. The cholesterol content was marginally higher in the diets containing fish oil (0·20–0·21 mg/g diet) compared with the control diet (0·18 mg/g diet) (Table 2).

**Table 2.** Contents of fatty acids and cholesterol in the diets

	Control diet	HERO diet	ANCO diet
Fatty acids (g/100 g diet)			
C14:0	0·02	0·26	0·12
C15:0	<LOQ	0·02	0·01
C16:0	0·92	0·95	0·98
C17:0	0·01	0·01	0·01
C18:0	0·31	0·24	0·30
C20:0	0·02	0·02	0·02
C22:0	0·02	0·02	0·02
C16:1 *n*-7	0·01	0·13	0·11
C18:1 *n*-11	<LOQ	0·04	0·03
C18:1 *n*-9	1·71	1·31	1·51
C18:1 *n*-7	0·12	0·11	0·13
C18:1 *n*-5	<LOQ	0·01	0·01
C20:1 *n*-11	<LOQ	0·05	<LOQ
C20:1 *n*-9	0·01	0·35	0·02
C22:1 *n*-11	<LOQ	0·70	<LOQ
C22:1 *n*-9	<LOQ	0·03	<LOQ
C24:1 *n*-9	<LOQ	0·03	<LOQ
C16:2 *n*-4	<LOQ	0·02	0·02
C16:3 *n*-4	<LOQ	0·01	0·02
C18:2 *n*-6	4·52	2·92	3·73
C18:3 *n*-3	0·57	0·39	0·48
C18:4 *n*-3	<LOQ	0·07	0·04
C20:5 *n*-3	<LOQ	0·17	0·23
C22:5 *n*-3	<LOQ	0·02	0·02
C22:6 *n*-3	<LOQ	0·18	0·14
Cholesterol (mg/100 g diet)	17·5	21·2	20·1

HERO, herring oil; ANCO, anchovy oil; LOQ, level of quantification.

The following fatty acids were below LOQ in all diets: C12:0, C23:0, C24:0, C14:1 *n*-5, C16:1 *n*-9, C17:1 *n*-7, C17:1 *n*-8, C18:1 *n*-9t, C20:1 *n*-7, C22:1 *n*-7, C18:2 *n*-6tc, C18:3 *n*-6, C18:2 *n*-4, C20:2 *n*-6, C20:3 *n*-6, C20:4 *n*-6, C20:3 *n*-3, C20:4 *n*-3, C21:5 *n*-3, C22:4 *n*-6 and C22:5 *n*-6.

### Feed and water intake

The energy intake and the water intake were measured 2–3 d before euthanisation and were similar between the groups (*P* ANOVA 0·65 and 0·88, respectively, Table 3). The daily water intake was very high for all rats, reflecting the established diabetes in the rats, and corresponded to 60–65 % of the rats’ body weight. For comparison, based on random registrations, the daily water intake was approximately 20 g per rat when fed the high-fat diet before the onset of diabetes. The estimated loss of glucose and nitrogen as carbamide in urine was high in all experimental groups near the end of the intervention period, with no differences between the groups (*P* ANOVA 0·96 and 0·69, respectively, Table 3).

**Table 3.** Feed and water intake registered during 24 h single housing and estimated loss of glucose and carbamide in urine (mean values and standard deviations)

	Control group		HERO group		ANCO group		*P* ANOVA
	Mean	sd	Mean	sd	Mean	sd	
Feed intake (kJ/24 h)	922	179	863	126	849	109	0·65
Water intake (g/24 h)	309	60	292	40	300	61	0·88
Estimated loss of glucose in urine (g/24 h)	32·3	7·7	31·4	5·3	32·3	5·6	0·96
Estimated loss of carbamide in urine (g/24 h)	3·6	0·8	3·4	1·0	3·9	0·9	0·69

HERO, herring oil; ANCO, anchovy oil.

Data are presented as mean and standard deviation for *n* 6 rats in each experimental group. Groups are compared using one-way ANOVA followed by Tukey HSD *post hoc* test when appropriate. *P* < 0·05 was considered significant.

### Body weight and blood glucose

The mean body weight was 481 (s d 36) g, with a mean non-fasting blood glucose of 6·6 (sd 0·8) mmol/l when the twenty-four rats were introduced to the high-fat diet. Of the twenty-four rats, eighteen developed diabetes (blood glucose > 13·9 mmol/l); that is, 75 % of the rats responded to the high-fat diet (25·4 kJ/g). The remaining six rats were excluded from the experiment. To maintain social stability, rats were kept as the original pairs all through the intervention period from the time of arrival at the animal house/facility till euthanisation. When diabetes was established, which was defined as the baseline for this study, the rats’ diets were gradually changed to one of the three experimental diets with regular energy content (19 kJ/g). The experimental groups were similar with regard to body weight and blood glucose concentration at baseline (*P* ANOVA 0·80 and 0·38, respectively, Table 4). As a result of overt diabetes and the change from a high-fat diet to a regular calorie diet, all rats lost weight during the intervention period. At the endpoint, no differences were seen between the experimental groups for body weight, body weight:square body length ratio, fasting blood glucose concentration or relative weights of liver and epididymal white adipose tissue (*P* ANOVA 0·24, 0·97, 0·97, 0·36 and 0·67, respectively, Table 4).

**Table 4.** Body weight and glucose at baseline (when diabetes was established and intervention started) and endpoint, change in body weight, body weight:square body length ratio and relative weights of liver and epididymal white adipose tissue (mean values and standard deviations)

	Control group		HERO group		ANCO group		
	Mean	sd	Mean	sd	Mean	sd	*P* ANOVA
Body weight at baseline (g)	539	40	537	35	550	37	0·80
Non-fasting blood glucose at baseline (mmol/l)	22·1	4·4	21·6	4·7	19·0	2·4	0·38
Body weight (g) at endpoint	474	23	465	24	488	22	0·24
Change in body weight (g)	–65	27	–72	15	–62	24	0·74
Body weight to square body length ratio (kg/m^2^) at endpoint	7·2	0·4	7·2	0·4	7·2	0·4	0·97
Fasting blood glucose at endpoint (mmol/l)	27·4	2·6	27·2	2·9	26·9	3·9	0·97
Relative liver weight (g/kg body weight) at endpoint	37·2	1·7	38·5	1·6	38·1	1·1	0·36
Relative WATepi weight (g/kg body weight) at endpoint	9·3	1·1	9·7	0·5	8·9	2·3	0·67

HERO, herring oil; ANCO, anchovy oil; WATepi, white adipose tissues.

Data are presented as mean and standard deviation for *n* 6 rats in each experimental group. Groups are compared using one-way ANOVA followed by Tukey HSD *post hoc* test when appropriate. *P* < 0·05 was considered significant.

### Serum analyses

**Fig. 1.**
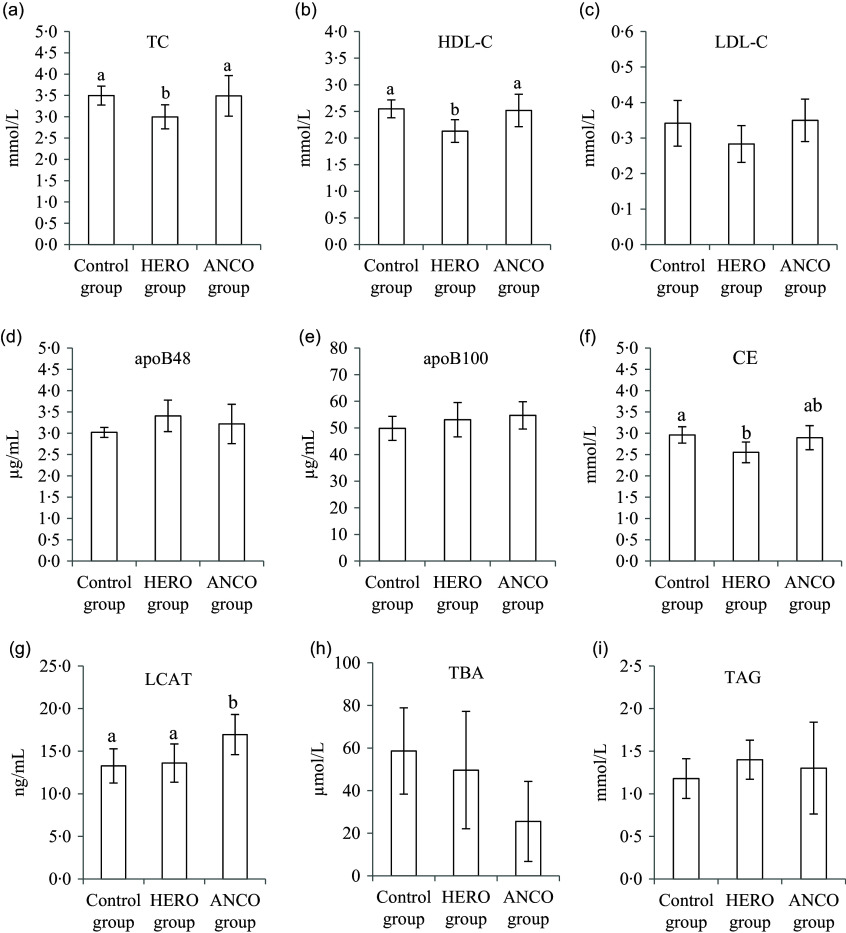
Serum concentrations of total cholesterol (TC) (a), HDL-cholesterol (b), LDL-cholesterol (c), apoB48 (d), apoB100 (e), cholesteryl esters (CE) (f), lecithin-cholesterol acyltransferase (LCAT) (g), total bile acids (TBA) (h) and TAG (i). Data are presented as mean and standard deviation for *n* 6 rats in each experimental group. Groups are compared using one-way ANOVA followed by Tukey HSD *post hoc* test when appropriate. Bars with different letters are significantly different (*P* < 0·05). HERO, herring oil; ANCO, anchovy oil.

Serum concentrations of total cholesterol and HDL-cholesterol were lower in the HERO group compared with both the Control group and ANCO group (*P* ANOVA 0·023 and 0·013, respectively, Figs. 1(a) and (b)), whereas the LDL-cholesterol concentration was similar in all groups (*P* ANOVA 0·14, Fig. 1(c)). Concentrations of apoB48 and apoB100 were also similar between the groups (*P* ANOVA 0·19 and 0·31, respectively, Figs. 1(d) and (e)). The concentration of cholesteryl ester was lower in the HERO group compared with the Control group (*P* ANOVA 0·023, Fig. 1(f)). The LCAT concentration was higher in the ANCO group compared with both the Control group and the HERO group (*P* ANOVA 0·021, Fig. 1(g)). The serum concentrations of total bile acid and TAG were similar between the groups (*P* ANOVA 0·070 and 0·58, respectively, Figs. 1(h) and (i)).

### Liver analyses

**Fig. 2.**
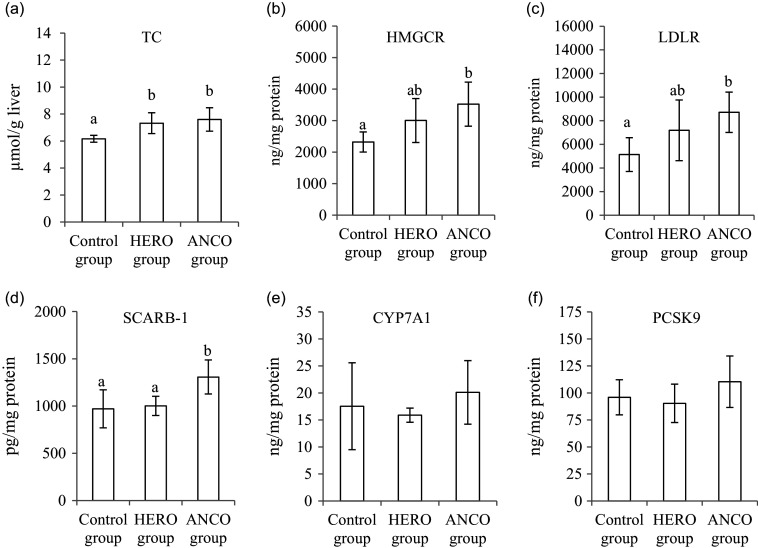
Liver concentrations of total cholesterol (TC) (a), HMG-CoA reductase (HMGCR) (b), LDL receptor (LDLR) (c), scavenger receptor class B, member 1 (SCARB-1) (d), cholesterol 7 *α*-hydroxylase (CYP7A1) (e) and proprotein convertase subtilisin/kexin type 9 (PCSK9) (f). Data are presented as mean and standard deviation for *n* 6 rats in each experimental group. Groups are compared using one-way ANOVA followed by Tukey HSD *post hoc* test when appropriate. Bars with different letters are significantly different (*P* < 0·05). HERO, herring oil; ANCO, anchovy oil.

The rats in both the HERO group and the ANCO group had higher liver cholesterol content when compared with the Control group (*P* ANOVA 0·0057, Fig. 2(a)). The hepatic concentrations (relative to protein) of HMG-CoA reductase (the rate-determining enzyme for the hepatic synthesis of cholesterol), LDL receptor and SCARB-1 were similar between the HERO group and the Control group, whereas the concentrations of all three were higher in the ANCO group when compared with the Control group (*P* ANOVA 0·012, 0·021 and 0·0055, respectively, Figs. 2(b)–(d)). The relative hepatic concentration of CYP7A1 (the rate-determining enzyme for the conversion of cholesterol to bile acids) was similar in all experimental groups (*P* ANOVA 0·47, Fig. 2(e)). The relative liver concentration of PCSK9, which is secreted from the liver and binds to the LDL receptor and reduces the clearance of LDL, was similar between the groups (*P* ANOVA 0·26, Fig. 2(f)).

**Fig. 3.**
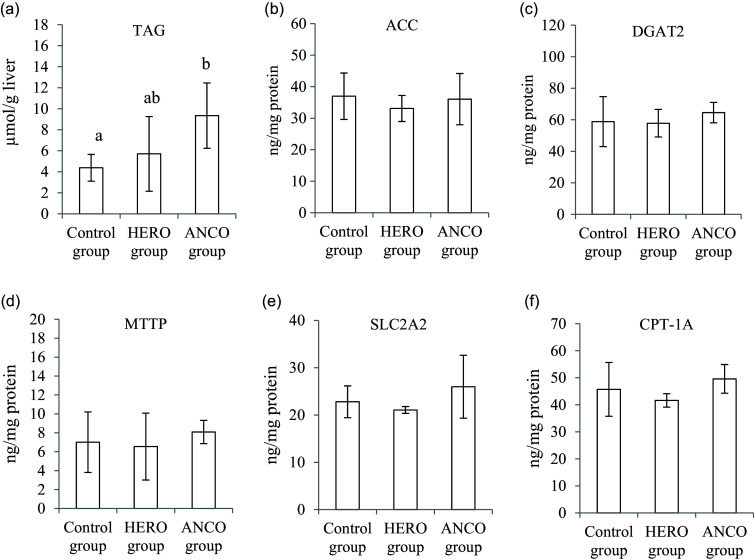
Liver concentrations of TAG (a), acetyl-CoA carboxylase (ACC) (b), diacylglycerol O-acyltransferase 2 (DGAT2 (c), microsomal triglyceride transfer protein (MTTP) (d), solute carrier family 2 (facilitated GLUT), member 2 (SLC2A2) (e) and carnitine palmitoyltransferase-1A (CPT-1A) (f). Data are presented as mean and standard deviation for *n* 6 rats in each experimental group. Groups are compared using one-way ANOVA followed by Tukey HSD *post hoc* test when appropriate. Bars with different letters are significantly different (*P* < 0·05). HERO, herring oil; ANCO, anchovy oil.

The hepatic TAG content was similar in the HERO group and the Control group but was higher in the ANCO group (*P* ANOVA 0·014, Fig. 3(a)). ACC, which catalyses the first committed step in the fatty acid synthesis; DGAT2, which catalyses the final step in the TAG synthesis and regulates VLDL production; and MTTP, which catalyses the lipidation of apoB100, were found in similar amounts in all groups (relative to protein, *P* ANOVA 0·59, 0·50 and 0·64, respectively, Figs 3(b)–(d)). The groups were similar with regard to the hepatic amounts of the major hepatic glucose transporter SLC2A2 and of CPT-1A, which is the rate-determining enzyme in fatty acid beta-oxidation by converting long-chain acyl-CoA into acyl-carnitine, thus allowing fatty acids to enter mitochondria for oxidation (*P* ANOVA 0·17 and 0·15, respectively, Figs 3(e) and (f)).

### Analyses in faeces

**Fig. 4.**
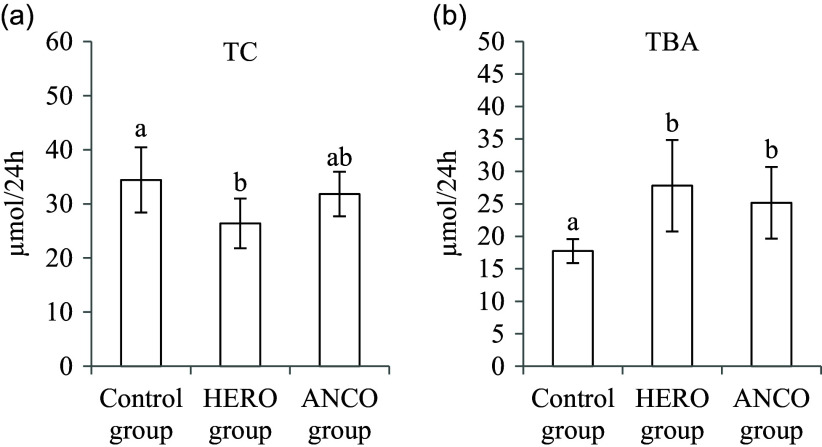
Faecal excretion of cholesterol (TC) (a) and bile acids (TBA) (b). Data are presented as mean and standard deviation for *n* 6 rats in each experimental group. Groups are compared using one-way ANOVA followed by Tukey HSD *post hoc* test when appropriate. Bars with different letters are significantly different (*P* < 0·05). HERO: herring oil; ANCO: anchovy oil.

The daily faecal output of cholesterol was lower in the HERO group compared with the Control group (*P* ANOVA 0·038, Fig. 4(a)), with no difference between the ANCO group and the Control group. The total daily faecal bile acid excretion was higher in both the HERO group and the ANCO group when compared with the Control group (*P* ANOVA 0·0034, Fig. 4(b)), with no difference between the HERO group and the ANCO group.

### Lipids in the kidney and heart

**Fig. 5.**
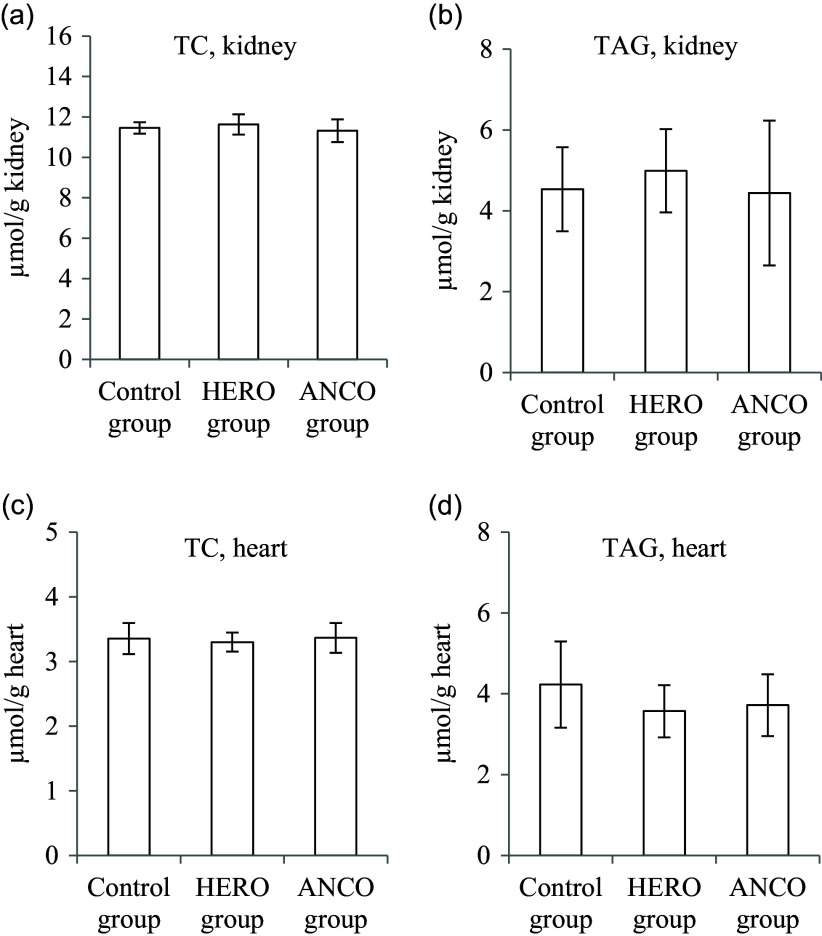
Lipids contents in tissues, showing total cholesterol (TC) in the kidney (a), TAG in the kidney (b), TC in the heart (c) and TAG in the heart (d). Data are presented as mean and standard deviation for *n* 6 rats in each experimental group. Groups are compared using one-way ANOVA, with the cutoff value for statistical significance set at a probability of 0·05. No differences were seen between the groups. HERO, herring oil; ANCO, anchovy oil.

To investigate if the lipid contents of other tissues besides the liver were affected by the HERO or the ANCO diets, we quantified the cholesterol and TAG contents in the kidney and heart. We found no differences between the groups for either lipid in these tissues (Fig. 5(a)–(d)).

## Discussion

In the present article, we show that when fed to diabetic ZDSD rats, a diet with fish oil-containing cetoleic acid (the HERO diet) resulted in a lower cholesterol concentration, whereas consumption of a diet containing fish oil with similar EPA + DHA content but without cetoleic acid (the ANCO diet) had no effect. Both experimental groups were compared with a Control group fed a diet devoid of fish oil. This is in line with our conclusion in a recent systematic review with meta-analysis investigating the effects of fish oil or fish oil concentrates with high cetoleic content fed to rodents^(23)^, although other fatty acids besides cetoleic acid, either from the diet or modified endogenously, may be responsible for this effect. Now we show for the first time that fish oils with or without cetoleic acid but with similar EPA + DHA content have markedly different effects on central enzymes and receptors involved in the catabolism and anabolism of cholesterol in the liver of diabetic rats. The findings suggest that the lower serum cholesterol concentration in rats fed the HERO diet was a result of a more efficient removal of bile acid in faeces, with no indication of effects on the hepatic concentrations of HMG-CoA reductase, LDL receptor or SCARB-1. The ANCO diet, on the other hand, did not affect the serum cholesterol concentration, but rats consuming this diet had higher hepatic concentrations of HMG-CoA reductase, LDL receptor and SCARB-1, as well as higher faecal output of bile acids.

The circulating cholesterol concentration is affected by many processes including uptake of dietary cholesterol, endogenous cholesterol synthesis in the liver followed by secretion of VLDL, removal from circulation by the liver and extrahepatic tissues, as well as excretion and reabsorption of cholesterol and bile acids in bile. In the present study, the cholesterol content was 15–21 % higher in the HERO diet and the ANCO diet when compared with the Control diet. The higher cholesterol intake was not reflected by a higher serum cholesterol concentration in either of the intervention groups when compared with controls but might partially explain the higher hepatic cholesterol content seen in both HERO and ANCO groups. On the contrary, the total cholesterol serum concentration was lower in rats fed the HERO diet and was similar in the ANCO group and the Control group.

The HERO diet did not affect the hepatic protein concentration of HMG-CoA reductase, which is the rate-determining enzyme for endogenous cholesterol synthesis and the primary target for cholesterol-lowering drugs^(36)^. This is in contrast to a study where male C57BL/6J mice fed a high-fat diet containing 15 % pollock oil (1·85 g cetoleic acid/100 g diet) and 17 % lard had a lower gene expression of HMG-CoA reductase (relative to 18S rRNA) compared with a high-fat diet with 32 % lard^(37)^. This discrepancy may be explained by the higher lard intake in the Control group compared with the pollock oil diet in the Yang 2011 study^(37)^ since palmitic acid (C16:0) increased HMG-CoA reductase mRNA and activity in HepG2 cells^(38)^ and oleic acid (C18:1n-9) was shown to stimulate HMG-CoA reductase activity in perfused rat liver^(39)^.

The rats in the HERO group and in the Control group were similar with regard to the hepatic concentrations of ACC, DGAT2, MTTP, CPT-1A and TAG, indicating that neither the fatty acid synthesis, the TAG synthesis, the rate of VLDL assembly nor the mitochondrial beta-oxidation of fatty acids were affected by the HERO diet. The hepatic concentration of SLC2A2, the major glucose transporter in liver, was also similar between the groups, again indicating that the *de novo* lipogenesis from glucose was similar between the groups. This is further supported by the similar serum concentrations of TAG, apoB100 and LDL-cholesterol between the HERO group and the Control group, suggesting that the VLDL secretion was similar in these groups. The findings that the concentrations of LDL receptor, PCSK9 and SCARB-1 in the liver and the serum apoB48 concentration were similar between the HERO group and the Control group suggest that the endocytosis of VLDL remnants, chylomicron remnants and LDL, as well as the extraction of cholesteryl esters from HDL and LDL from the circulation were not affected by the HERO diet. Biliary excretion of cholesterol and conversion of cholesterol to bile acids are the principal routes of cholesterol excretion and catabolism. The faecal cholesterol excretion was lower in the HERO group, which may, at least in part, explain the higher hepatic cholesterol content due to inferior removal of cholesterol in the bile, but it may also reflect a higher uptake of dietary cholesterol followed by storage in the liver. Of great interest is that the faecal excretion of bile acids was markedly higher in the HERO group, and even with no effect on the hepatic CYP7A1 concentration, we suggest that low reabsorption of bile acids from the enterohepatic circulation leading to enhanced loss of bile acids is the main explanation for the lower serum cholesterol concentration in rats fed the HERO diet.

The ANCO diet did not affect the serum concentrations of total cholesterol, HDL- cholesterol and LDL-cholesterol when compared with the Control group. The higher liver concentrations of HMG-CoA reductase, LDL receptor and SCARB-1 indicate both a higher endogenous cholesterol production and enhanced uptake of lipoproteins to the liver, all contributing to the accumulation of cholesterol in the liver of rats fed the ANCO diet, as is supported by a higher liver cholesterol content. The higher SCARB-1 concentration in the liver of ANCO-fed rats is in line with elevated gene expression of SCARB-1 in female C57BL/6J mice fed a relatively high dose of EPA + DHA concentrate, causing a lower HDL-C concentration in the latter^(40)^. The ANCO group had a higher liver content of TAG when compared with the Control group, but the level was still well below the limit for fatty liver, which is defined as TAG content above 5 wt% (corresponding to approximately 60 µmol TAG/g liver). Liver ACC, DGAT2, MTTP, SLC2A2, CPT-1A and serum TAG concentrations were similar to those of the Control group, thus proposing that the *de novo* lipogenesis from glucose, TAG synthesis, VLDL assembly and mitochondrial beta-oxidation were not affected by the ANCO diet. In addition, ANCO-fed rats had a higher serum concentration of LCAT, which is bound to HDL and catalyses the transfer of an acyl group from phospholipids to free cholesterol to produce cholesteryl esters, resulting in a larger spherical HDL particle. The higher serum concentration of LCAT in the ANCO group indicates a more active esterification of cholesterol to produce cholesteryl esters and larger spherical HDL particles; however, no difference was seen between the ANCO group and the Control group with regard to the serum cholesteryl ester concentration. Analyses of the faecal excretion revealed no effect of the ANCO diet on cholesterol removal, whereas the bile acid excretion was markedly higher when compared with the Control group. Thus, the effects of elevated cholesterol biosynthesis in the liver, more hepatic cholesterol uptake from circulation and higher bile acid excretion in faeces evidently cancel each other out and result in no effect of the ANCO diet on the circulating cholesterol concentration.

The lipid contents were quantified in the kidneys and the heart, to investigate if the higher cholesterol content in the liver in the HERO and ANCO groups and the higher TAG content in the ANCO group were a general ‘organ effect’ after consumption of these diets. However, no differences were seen between the dietary groups for either cholesterol or TAG in these organs, thus suggesting that the effects of the diets seen in the lipids in the liver were results of differences in the hepatic lipid metabolism and not the result of a whole-body effect.

Lowering the serum cholesterol concentration is an important strategy for reducing the risk of CVD, and it is estimated that a reduction in total cholesterol concentration of 3 % provides a 15 % reduction in CHD risk^(41)^. In the present study, the mean serum total cholesterol concentration was 14 % lower in the rats fed the HERO diet compared with the Control group. HDL is the main cholesterol transporter in rodents, and the level of LDL cholesterol is in general very low. In the present study, HDL cholesterol constituted 71–73 %, and LDL cholesterol constituted around 10 % of the total cholesterol concentration, whereas in humans, cholesterol is mainly carried by LDL. This difference in cholesterol transport should be taken into account when findings in the present study are interpreted in relation to human cholesterol metabolism. The effect of the fish oil-containing diets on the distribution of HDL of different size-defined subclasses was not investigated in the present study. Large HDL particles have been proposed to be more anti-atherogenic compared with smaller-size HDL^(42)^, and analysis of the HDL size distribution would have provided valuable information that may potentially increase the relevance of the finding of a lower HDL-cholesterol concentration in rats fed the HERO diet for humans. Findings in rat studies are not directly transferable to humans; however, the present findings may be clinically relevant and should be further explored in humans with a high risk for hypercholesterolaemia.

The present study has some strength and limitations. Strengths include measurements of many central enzymes, receptors, transporters, regulators, substrates and metabolites involved in the cholesterol metabolism in the liver, serum and faeces from rats. Limitations to the study include the choice of animal model used, as the observed effects of a cetoleic acid-containing fish oil intake in rats with overt T2D may be specific to the ZDSD rats and must be further investigated through mechanistic studies using other animal models and also in both diabetic and non-diabetic humans. The fish oil-containing diets were balanced with regard to amounts of EPA + DHA; therefore, more HERO than anchovy oil was added to the diets, and this should be taken into account when comparing the effects of the respective diets. Additional knowledge regarding the accumulation of cetoleic acid and its possible metabolites as well as more information about how the amounts of fatty acids including EPA and DHA are affected in tissues is warranted, and this is currently under investigation in our research group.

### Conclusion

In the present study, we compare the effects of two diets with similar content of EPA + DHA from fish oils, one of which also contained cetoleic acid, with the aim to investigate their effect on markers of cholesterol metabolism in the liver. The two marine oils, that is, herring oil (containing cetoleic acid) and anchovy oil (virtually devoid of cetoleic acid), had remarkably different effects on the cholesterol metabolism in diabetic rats. Consumption of herring oil resulted in a lower cholesterol concentration through a higher faecal excretion of bile acids and without affecting the endogenous cholesterol production in the liver, the hepatic secretion of VLDL or the capacity for the liver to take up cholesterol from lipoproteins or lipoprotein remnants. Intake of anchovy oil did not affect the serum cholesterol concentration but resulted in a higher hepatic cholesterol biosynthesis, more clearance of lipoprotein cholesterol and elevated faecal excretion of bile acids. We conclude that consumption of a diet containing herring oil leads to a lower cholesterol concentration in a relevant rat model for T2D in humans.

## References

[1] Ingelsson E , Schaefer EJ , Contois JH , et al. (2007) Clinical utility of different lipid measures for prediction of coronary heart disease in men and women. JAMA 298, 776–785. 17699011 10.1001/jama.298.7.776

[2] Prospective Studies Collaboration (2007) Blood cholesterol and vascular mortality by age, sex, and blood pressure: a meta-analysis of individual data from 61 prospective studies with 55 000 vascular deaths. Lancet 370, 1829–1839. 18061058 10.1016/S0140-6736(07)61778-4

[3] Roth GA , Mensah GA , Johnson CO , et al. (2020) Global Burden of Cardiovascular Diseases and Risk Factors, 1990–2019: update from the GBD 2019 study. J Am Coll Cardiol 76, 2982–3021. 33309175 10.1016/j.jacc.2020.11.010 PMC7755038

[4] De Rosa S , Arcidiacono B , Chiefari E , et al. (2018) Type 2 diabetes mellitus and cardiovascular disease: genetic and epigenetic links. Front Endocrinol (Lausanne) 9, 2. 29387042 10.3389/fendo.2018.00002 PMC5776102

[5] Howard BV , Best LG , Galloway JM , et al. (2006) Coronary heart disease risk equivalence in diabetes depends on concomitant risk factors. Diabetes Care 29, 391–397. 16443893 10.2337/diacare.29.02.06.dc05-1299

[6] Emerging Risk Factors Collaboration, Sarwar N , Gao P , et al. (2010) Diabetes mellitus, fasting blood glucose concentration, and risk of vascular disease: a collaborative meta-analysis of 102 prospective studies. Lancet 375, 2215–2222. 20609967 10.1016/S0140-6736(10)60484-9 PMC2904878

[7] Davies MJ , D’Alessio DA , Fradkin J , et al. (2018) Management of hyperglycemia in type 2 diabetes, 2018. A consensus report by the American Diabetes Association (ADA) and the European Association for the Study of Diabetes (EASD). Diabetes Care 41, 2669–2701. 30291106 10.2337/dci18-0033 PMC6245208

[8] National Clinical Guideline Centre (UK) (2014) Lipid Modification: Cardiovascular Risk Assessment and the Modification of Blood Lipids for the Primary and Secondary Prevention of Cardiovascular Disease. London: National Institute for Health and Care Excellence (UK). 25340243

[9] Zheng J , Huang T , Yu Y , et al. (2012) Fish consumption and CHD mortality: an updated meta-analysis of seventeen cohort studies. Public Health Nutr 15, 725–737. 21914258 10.1017/S1368980011002254

[10] Virtanen JK , Mozaffarian D , Chiuve SE , et al. (2008) Fish consumption and risk of major chronic disease in men. Am J Clin Nutr 88, 1618–1625. 19064523 10.3945/ajcn.2007.25816 PMC2613199

[11] He K , Song Y , Daviglus ML , et al. (2004) Accumulated evidence on fish consumption and coronary heart disease mortality: a meta-analysis of cohort studies. Circulation 109, 2705–2711. 15184295 10.1161/01.CIR.0000132503.19410.6B

[12] Kromann N & Green A (1980) Epidemiological studies in the Upernavik district, Greenland. Incidence of some chronic diseases 1950–1974. Acta Med Scand 208, 401–406. 7457208

[13] Feskens EJ , Bowles CH & Kromhout D (1991) Inverse association between fish intake and risk of glucose intolerance in normoglycemic elderly men and women. Diabetes Care 14, 935–941. 1797505 10.2337/diacare.14.11.935

[14] Nkondjock A & Receveur O (2003) Fish-seafood consumption, obesity, and risk of type 2 diabetes: an ecological study. Diabetes Metab 29, 635–642. 14707894 10.1016/s1262-3636(07)70080-0

[15] Mozaffarian D & Wu JH (2011) *n*-3 fatty acids and cardiovascular disease: effects on risk factors, molecular pathways, and clinical events. J Am Coll Cardiol 58, 2047–2067. 22051327 10.1016/j.jacc.2011.06.063

[16] Eslick GD , Howe PR , Smith C , et al. (2009) Benefits of fish oil supplementation in hyperlipidemia: a systematic review and meta-analysis. Int J Cardiol 136, 4–16. 18774613 10.1016/j.ijcard.2008.03.092

[17] Harris WS (1997) *n*-3 fatty acids and serum lipoproteins: human studies. Am J Clin Nutr 65, 1645S–1654S. 9129504 10.1093/ajcn/65.5.1645S

[18] Balk EM , Lichtenstein AH , Chung M , et al. (2006) Effects of *n*-3 fatty acids on serum markers of cardiovascular disease risk: a systematic review. Atherosclerosis 189, 19–30. 16530201 10.1016/j.atherosclerosis.2006.02.012

[19] Innes JK & Calder PC (2018) The differential effects of eicosapentaenoic acid and docosahexaenoic acid on cardiometabolic risk factors: a systematic review. Int J Mol Sci 19, 532–553. 29425187 10.3390/ijms19020532 PMC5855754

[20] Harris WS (1997) *n*-3 fatty acids and serum lipoproteins: animal studies. Am J Clin Nutr 65, 1611S–1616S. 9129501 10.1093/ajcn/65.5.1611S

[21] Reinwald S , Peterson RG , Allen MR , et al. (2009) Skeletal changes associated with the onset of type 2 diabetes in the ZDF and ZDSD rodent models. Am J Physiol Endocrinol Metab 296, E765–E774. 19158319 10.1152/ajpendo.90937.2008 PMC2670632

[22] Wang AN , Carlos J , Fraser GM , et al. (2022) Zucker Diabetic-Sprague Dawley (ZDSD) rat: type 2 diabetes translational research model. Exp Physiol 107, 265–282. 35178802 10.1113/EP089947 PMC9314054

[23] Mjaatveit M , Oldernes H & Gudbrandsen OA (2024) Effects of diets containing fish oils or fish oil concentrates with high cetoleic acid content on the circulating cholesterol concentration in rodents. A systematic review and meta-analysis. Br J Nutr 131, 606–621. 37737066 10.1017/S0007114523002118 PMC10803824

[24] ZDSD Rat Diet Recommendations Strain Code: 696. Charles River Research Models. www.criver.com (accessed September 2022).

[25] Reeves PG , Nielsen FH & Fahey GC Jr (1993) AIN-93 purified diets for laboratory rodents: final report of the American Institute of Nutrition writing committee on the reformulation of the AIN-76A rodent diet. J Nutr 123, 1939–1951. 8229312 10.1093/jn/123.11.1939

[26] Reeves PG (1996) AIN-93 purified diets for the study of trace element metabolism in rodents. In Trace Elements in Laboratory Rodents, pp. 3–37 [ RR Watson , editor]. Boca Raton, FL: CRC Press Inc.

[27] Wang X , Hu Z , Hu J , et al. (2006) Insulin resistance accelerates muscle protein degradation: activation of the ubiquitin-proteasome pathway by defects in muscle cell signaling. Endocrinol 147, 4160–4168. 10.1210/en.2006-0251 16777975

[28] Abdulla H , Smith K , Atherton PJ , et al. (2016) Role of insulin in the regulation of human skeletal muscle protein synthesis and breakdown: a systematic review and meta-analysis. Diabetologia 59, 44–55. 26404065 10.1007/s00125-015-3751-0

[29] Gudbrandsen OA (2024) Periodic stays in a ‘playcage’ as an environmental enrichment measure for laboratory rats housed in individually ventilated cages: short report. Lab Anim (Epublication ahead of print version 25 July 2024). 10.1177/00236772231209198 39053475

[30] Bligh EG & Dyer WJ (1959) A rapid method of total lipid extraction and purification. Can J Biochem Physiol 37, 911–917. 13671378 10.1139/o59-099

[31] Drotningsvik A , Mjos SA , Hogoy I , et al. (2015) A low dietary intake of cod protein is sufficient to increase growth, improve serum and tissue fatty acid compositions, and lower serum postprandial glucose and fasting non-esterified fatty acid concentrations in obese Zucker fa/fa rats. Eur J Nutr 54, 1151–1160. 25380663 10.1007/s00394-014-0793-x

[32] International Organization for Standardization (1998) Animal Feeding Stuffs, Animal Products, and Faeces or Urine - Determination of Gross Calorific Value - Bomb Calorimeter Method (ISO 9831:1998). https://www.iso.org/standard/17702.html (accessed 19 March 2019).

[33] Bradford MM (1976) A rapid and sensitive method for the quantitation of microgram quantities of protein utilizing the principle of protein-dye binding. Anal Biochem 72, 248–254. 942051 10.1016/0003-2697(76)90527-3

[34] Suckling KE , Benson GM , Bond B , et al. (1991) Cholesterol lowering and bile acid excretion in the hamster with cholestyramine treatment. Atherosclerosis 89, 183–190. 1793446 10.1016/0021-9150(91)90059-c

[35] Lee S & Lee DK (2018) What is the proper way to apply the multiple comparison test? Korean J Anesthesiol 71, 353–360. 30157585 10.4097/kja.d.18.00242 PMC6193594

[36] Feingold KR & Grunfeld C (2000) Cholesterol lowering drugs. In Endotext [ LJ De Groot , G Chrousos , K Dungan , KR Feingold , A Grossman , JM Hershman , C Koch , M Korbonits , R McLachlan , M New , et al., editors]. South Dartmouth, MA: MDTEXT.COM.

[37] Yang ZH , Miyahara H , Takeo J , et al. (2011) Pollock oil supplementation modulates hyperlipidemia and ameliorates hepatic steatosis in mice fed a high-fat diet. Lipids Health Dis 10, 189–198. 22027268 10.1186/1476-511X-10-189 PMC3215994

[38] Wu N , Sarna LK , Hwang SY , et al. (2013) Activation of 3-hydroxy-3-methylglutaryl coenzyme A (HMG-CoA) reductase during high fat diet feeding. Biochim Biophys Acta 1832, 1560–1568. 23651731 10.1016/j.bbadis.2013.04.024

[39] Salam WH , Wilcox HG , Cagen LM , et al. (1989) Stimulation of hepatic cholesterol biosynthesis by fatty acids. Effects of oleate on cytoplasmic acetoacetyl-CoA thiolase, acetoacetyl-CoA synthetase and hydroxymethylglutaryl-CoA synthase. Biochem J 258, 563–568. 2565110 10.1042/bj2580563 PMC1138398

[40] le Morvan V , Dumon MF , Palos-Pinto A , et al. (2002) *n*-3 FA increase liver uptake of HDL-cholesterol in mice. Lipids 37, 767–772. 12371747 10.1007/s11745-002-0959-2

[41] Fager G & Wiklund O (1997) Cholesterol reduction and clinical benefit. Are there limits to our expectations? Arterioscler Thromb Vasc Biol 17, 3527–3533. 9437202 10.1161/01.atv.17.12.3527

[42] Kontush A (2015) HDL particle number and size as predictors of cardiovascular disease. Front Pharmacol 6, 218. 26500551 10.3389/fphar.2015.00218 PMC4593254

